# EASE-CF: dietitian-led weight-loss intervention for adults with cystic fibrosis and excess weight: protocol for a feasibility randomised controlled trial

**DOI:** 10.1136/bmjresp-2026-004328

**Published:** 2026-07-27

**Authors:** Joanna Snowball, Amanda Adler, Phillippa Lally, Nick P Talbot, Emma Hedley, Dimitrios A Koutoukidis

**Affiliations:** 1 Nuffield Department of Primary Care Health Sciences, University of Oxford, Oxford, UK; 2 Oxford Centre for Respiratory Medicine, Oxford University Hospitals NHS Foundation Trust, Oxford, UK; 3 Radcliffe Department of Medicine, University of Oxford, Oxford, UK; 4 School of Psychology, University of Surrey, Guildford, UK; 5 Department of Physiology, Anatomy and Genetics, University of Oxford, Oxford, UK; 6 Oxford Respiratory Trials Unit, University of Oxford, Oxford, UK

**Keywords:** Cystic Fibrosis

## Abstract

**Introduction:**

Advances in cystic fibrosis (CF) care have coincided with an increasing prevalence of excess body weight in this population, which may contribute to cardiometabolic and respiratory morbidity. However, evidence to guide intentional weight loss in this population is limited and safety concerns remain due to the established positive association between body weight and lung function. Delivering weight-loss interventions in CF is likely to be complex because of CF-specific factors, high treatment burden and embedded dietary advice focused on high-energy, high-fat diets for weight gain. To inform the design of a future definitive randomised controlled trial (RCT), we plan to test the feasibility, acceptability and safety of a dietitian supported weight-loss intervention compared with routine care.

**Methods and analysis:**

This multicentre feasibility RCT with an embedded qualitative study, will recruit 30 adults with CF and body mass index ≥27 kg/m^2^ from four UK specialist centres. Participants will be randomised 2:1 to the EASE-CF intervention or routine dietetic consultations, with follow-up over 24 weeks. The remotely delivered, theory-informed intervention combines structured dietary energy restriction, meal replacements and behavioural support from specialist CF dietitians. Participants randomised to routine care will continue to receive standard CF dietetic input. The primary objective is to assess the feasibility of progression to a definitive RCT based on pre-specified criteria on recruitment, engagement, adherence, retention and safety. Assessment of intervention acceptability and fidelity will be explored through process measures and qualitative interviews with participants and healthcare professionals. Exploratory outcomes include changes in body weight and composition, lung function, quality of life, physical function and metabolic markers.

**Ethics and dissemination:**

Ethical approval has been granted by the National Research Ethics Service South Central B Research Ethics Committee (ref: 25/SC/0323). Findings will be disseminated through stakeholder engagement, conference presentations and publication in open-access, peer-reviewed journals.

**Trial registration number:**

ISRCTN17298282.

**WHAT IS ALREADY KNOWN ON THIS TOPIC**

- A growing proportion of adults with cystic fibrosis (CFNor) now live with overweight or obesity. However, structured weight reduction strategies have not been formally evaluated in CF, and clinicians lack evidence to guide safe management of excess weight in this context.

**WHAT THIS STUDY ADDS**

- This study evaluates the feasibility of delivering a specialist dietitian-led weight loss intervention for adults with CF and excess weight, including recruitment, retention, engagement and adherence. An embedded qualitative process evaluation will examine intervention acceptability, fidelity and implementation challenges.

**HOW THIS STUDY MIGHT AFFECT RESEARCH, PRACTICE OR POLICY**

- Remote delivery of the theory-informed intervention reflects current clinical practice, reduces participant burden and supports scalability within routine CF care. As a feasibility study, it is not powered to assess clinical effectiveness. Findings will inform the design of a definitive randomised controlled trial.

## Introduction

 Cystic fibrosis (CF) is a rare, genetic, life-limiting condition characterised by progressive lung disease and multisystem involvement, requiring complex multidisciplinary care.^1^ Advances in treatment have improved survival, with UK registry data showing the median life expectancy increasing from 36 years in 2013 to an estimated 64 years in 2024.^2^ These gains are largely attributable to the introduction of CF transmembrane conductance regulator (CFTR) modulators, which improve the function of the CFTR protein and are prescribed according to genotype.^3^ On average, CFTR modulator therapy is associated with improved lung function, reduced frequency of infective pulmonary exacerbations and enhanced quality of life.^4^ Consequently, CF services are now adapting to the needs of a heterogeneous and ageing adult population.^5^

Improved survival has been accompanied by important shifts in nutritional status. Historically, CF was associated with undernutrition, and dietary guidance promoted high-energy, high-fat intake (the ‘CF legacy diet’).^6^ However, excess weight (body mass index (BMI) ≥25 kg/m²) now affects over 40% of UK adults with CF (54% of those aged >50 years), three times the prevalence of underweight, making this approach unsuitable for many and potentially contributing to further weight gain.^2^

The mechanisms underlying weight gain on CFTR modulators include reduced total energy expenditure, improved appetite and enhanced nutrient absorption due to changes in gut function.^7^ Although the prevalence of overweight and obesity in people with CF remains lower than in the general population, this trend is concerning because people with CF have an increased risk of cardiovascular disease and gastrointestinal cancers compared with the general population, and excess weight may further compound these risks.^8 9^ Emerging evidence also suggests that excess weight may adversely affect respiratory health, including lung function decline.^10 11^

Weight loss may confer benefits similar to those observed in other chronic conditions.^12^ However, there is limited trial-based CF-specific evidence examining the rate, magnitude or safety of weight reduction, with concerns including unintended loss of muscle mass and deterioration in lung function. Published case reports describing bariatric surgery or GLP-1 receptor agonist therapy for weight loss in people with CF have raised concerns about physical fitness and function that warrant further detailed evaluation.^13–15^

Achieving intentional weight loss in people with CF presents specific challenges. Long-standing guidance for the ‘CF legacy diet’ may create cognitive dissonance when energy restriction is advised. Consequently, individuals with CF may need to modify ingrained eating habits, often established in childhood and reinforced during adulthood by clinicians and their social, cultural and economic environments.^16^ In addition, CF-specific complications including pancreatic insufficiency, gastrointestinal complications and CF-related diabetes need to be considered when dietary changes are introduced. The high daily clinical treatment burden may also limit engagement with structured weight-loss interventions. Collectively, these factors suggest that weight-loss interventions in CF may need to target the behavioural processes required to establish and sustain dietary change.

Evidence from weight-loss trials conducted in other chronic disease populations can inform intervention design, but these approaches require testing in a CF-specific context. In the UK, dietetic support is embedded within CF services, and both patients and clinicians view dietitian supported weight loss support as appropriate. However, uncertainty remains regarding the feasibility, acceptability and optimal delivery of weight-loss interventions within routine CF care.^16–18^

Despite the increasing prevalence of excess weight in CF, no clinical trials have evaluated intentional weight loss in this population, and current CF nutrition guidelines provide no structured recommendations for assessment or management of overweight and obesity.^19–21^ This reflects ongoing clinical equipoise, limited CF-specific evidence and practical constraints within CF teams, highlighting the need for feasibility testing prior to a definitive trial. The importance of addressing this evidence gap is further highlighted by the 2023 James Lind Alliance Priority Setting Partnership that identified the question “How can overweight and obesity be effectively managed in people with CF?” within its fourth highest-ranked research priority of “How do we manage an ageing population with CF?”.^22^

In response to these uncertainties, we developed a theory-informed weight-loss intervention with input from CF clinicians and people with lived experience of CF and excess weight. The intervention combines structured dietary energy restriction, including meal replacements and ready-prepared meals, with behavioural support delivered by specialist CF dietitians.

We have planned a randomised controlled feasibility trial to assess whether a definitive trial is warranted. Primary feasibility outcomes include recruitment and willingness to be randomised, engagement and adherence to the intervention, retention, acceptability of the intervention and study procedures, and safety, including potential effects on CF-specific health outcomes such as lung function. Qualitative interviews with participants and clinicians will explore acceptability and implementation processes to inform optimisation of the intervention and trial design.^23^

## Methods and analysis

### Study design and setting

This feasibility study is a randomised controlled trial (RCT) in adults with CF and a BMI ≥27 kg/m^2^, recruited from specialist CF centres in the UK. Each participant will be enrolled for 24 weeks from randomisation to final follow-up and will attend four study assessments. Due to the nature of the intervention, it will not be possible to blind the participants or clinicians delivering the intervention.

### Recruitment

Participants will be recruited from four UK CF centres over a period of 12 months; centres were identified via an expression of interest and feasibility assessment coordinated by the UK CF Trust Clinical Trials Accelerator Platform team. Sites were selected based on dietetic and research staff capacity and geographical representation. Established patient cohorts enable database screening to identify potentially eligible participants. Reasons for declining participation will be recorded using a predefined list in the screening log to inform assessment of recruitment feasibility.

### Inclusion criteria

Key inclusion criteria include:

- Aged ≥18 years.
- BMI ≥27 kg/m^2^ (or BMI ≥25 kg/m^2^ for people of Black, Asian or minority ethnic origin).^24^ The threshold of ≥27 kg/m² was selected to minimise the risk of participants reducing their weight below recommended CF BMI targets (≥23 kg/m²) during the intervention weight loss, and to avoid inclusion of individuals with mild overweight who may be more likely to fall below these thresholds. This cut-off also aligns with CF nutritional guidelines that define overweight at BMI ≥27 kg/m²).^19^
- Access to the internet and an internet-enabled device (smartphone, tablet, computer – a device can be provided if required).
- Established diagnosis of CF including those who have previously received a lung or liver transplant.
- Forced expiratory volume (FEV1) ≥25% predicted recorded in the last 6 months.

### Exclusion criteria

Key exclusion criteria include:

- Currently participating in a structured weight-loss intervention or ≥10% self‐reported weight loss in the 6 months before the screening visit.
- Documented decompensated liver disease.
- Documented stage 4–5 chronic kidney disease.
- Actively using enteral feeding.
- Pregnancy or breastfeeding.

### Study procedures and participant flow

**Figure 1:**
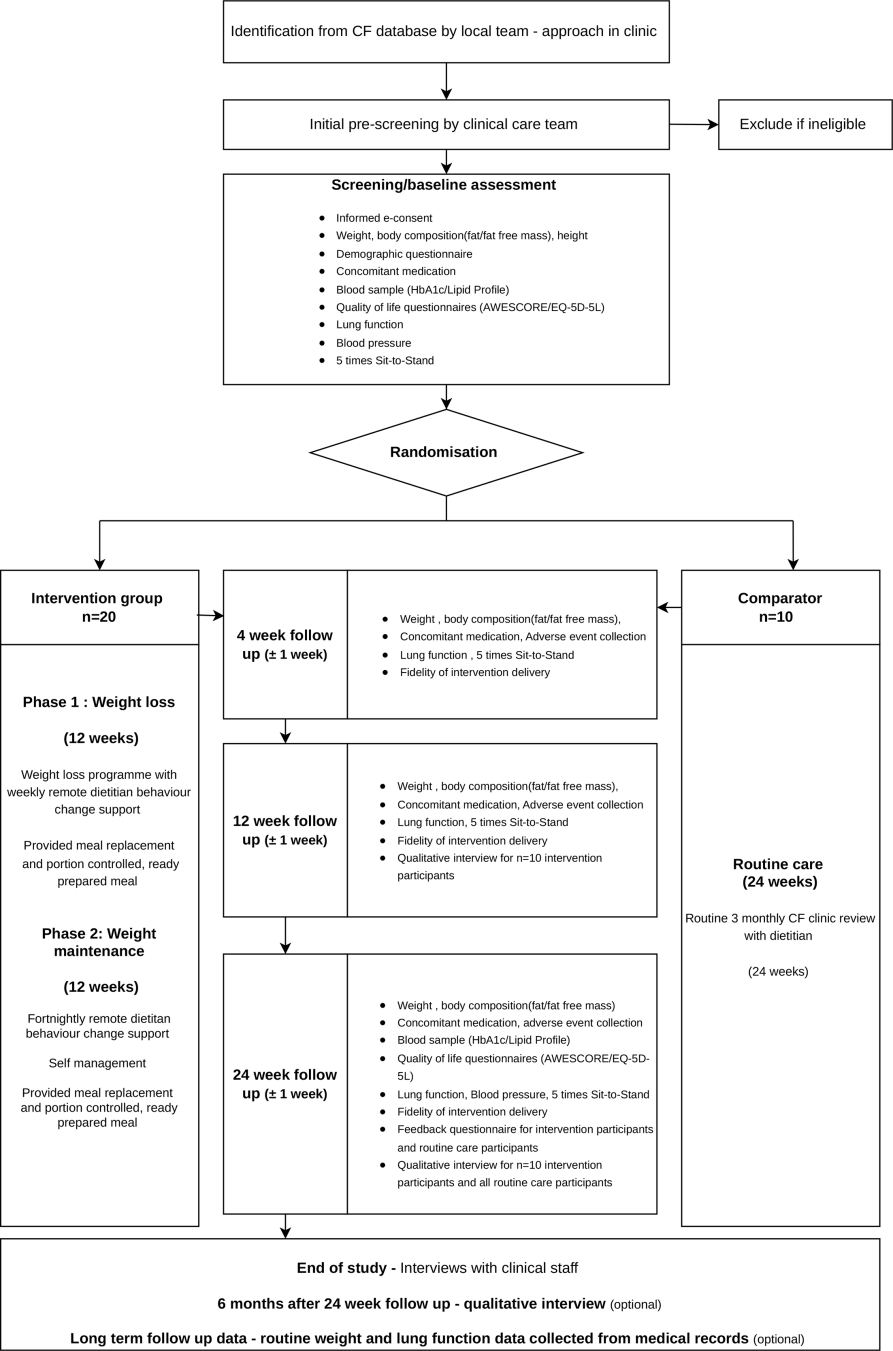
Participant flow through the study. CF, cystic fibrosis; EQ-5D-5L, EuroQol 5-dimension 5-level.

All study visits will be conducted remotely via Microsoft Teams or equivalent to minimise participant burden and assess the feasibility of home measurements. The baseline assessment will last approximately 60 min and follow-up assessments 15–20 min. Technical support to help participants access Microsoft Teams will be provided where required. Informed consent will be obtained remotely by a delegated member of the local research team. Eligibility will then be confirmed, and baseline assessments will be conducted using home monitoring equipment posted to the participant prior to the baseline assessment and a returned blood sample kit. Once all baseline measurements are finalised, participants will be randomised. Those allocated to the routine care group will follow the local standard care pathway attending their CF appointments. Participants in the intervention group will attend an initial dietetic consultation to receive intervention materials and commence the intervention. Remote assessments will take place at 4, 12 and 24 weeks. Intervention participants will participate in a qualitative interview at 12 or 24 weeks, and routine care participants will have a qualitative interview at 24 weeks. Weight and lung function data will be obtained from routine clinical records, including data collected as part of standard care and reported to the UK CF Registry, for up to 2 years post-study. Participant flow is shown in figure 1.

### Sample size

The study aims to recruit 30 participants (20 intervention, 10 routine care). As this is a feasibility study, it is not powered to detect a clinically or statistically significant difference between groups.^25^ The sample size was not arrived at using a statistical method but was informed by guidance to estimate key feasibility criteria with sufficient precision to inform progression decisions and the design of a definitive trial.^26^ The 2:1 allocation ratio allows greater experience of the intervention while retaining a comparator group to assess feasibility processes.

### Randomisation

All eligible and consented participants will be randomised 2:1 into the intervention group or routine care using a random permuted block design with blocks of 3 and 6, stratified by presence of CF-related diabetes (yes/no) and age (> or ≤40 years). A researcher independent of the study will generate the allocation sequence using the Sealed Envelope website,^27^ and a member of the local research team will enter the participant details into the randomisation system (Sealed Envelope) but will not have access to the allocation sequence, ensuring allocation concealment. The system will then automatically allocate the participant. Participants will then be informed of their allocation by the local research team. Blinding of participants and dietitians is not possible due to the nature of the intervention; however, risk of bias will be reduced through objective outcomes and standardised data collection.

### Intervention: the EASE-CF programme

EASE-CF is a structured weight-loss programme delivered by a specialist CF dietitian to support adults with CF and excess weight to achieve weight loss of 5%–10% over a 24 week period. The intervention combines structured energy restriction (including a meal replacement product and a portion-controlled ready-prepared meal), individualised goal setting and accountability to a dietitian to promote dietary behaviour change, habit formation and increased self-efficacy.

As, to our knowledge, no previous trials of weight-loss programmes for people with CF have been conducted, the intervention was informed by evidence from other chronic health conditions, relevant study protocols and CF clinician input, including a previous qualitative study exploring CF clinicians’ views on this topic^16^ and a survey of current dietetic practice for overweight and obesity.^17^ A group of people with lived experience of CF and excess weight also helped to shape the research question and inform the mode, delivery and length of the intervention.

The programme includes an initial 60 min consultation to assess dietary patterns and social circumstances, introduce the programme and establish personalised goals, followed by weekly 20 min video consultations to review progress, support self-monitoring of weight, reinforce behaviour change strategies, address barriers, provide accountability and facilitate action planning. The frequency of sessions may be reduced to fortnightly, based on mutual agreement between the dietitian and the participant if the participant is progressing sufficiently towards their weight loss target. Participants continue routine CF dietetic care as these include assessment of other clinical issues not included in the EASE-CF programme. Dietitians delivering the intervention are registered dietitians working within specialist CF services. All study dietitians receive a 2-hour study-specific training session prior to delivering the intervention covering the intervention protocol, behaviour change components and delivery procedures, together with a detailed programme manual and access to central support where required. The dietary component comprises three eating occasions per day (~1300 kcal/day). This standardised prescription was selected to maximise intervention fidelity and simplify delivery within this feasibility study. Participants are monitored throughout the intervention, and dietary adjustments may be made where necessary based on weight loss progress, tolerability and clinical considerations. Each day participants will be asked to consume: (1) A meal replacement product (shake/porridge of 200 kcal, Habitual Healthcare), (2) A portion-controlled high-protein ready meal of up to 600 kcal (Frive) and (3) An additional meal chosen by the participant based on dietitian’s advice for healthful eating. Water or no/low-calorie drinks are advised, with up to two snacks (one high-protein energy-restricted snack and one portion of fruit). The meal replacement products and ready meals are purchased using study funding and provided to participants free of charge. The manufacturers have no role in the study design, data collection, analysis, interpretation or publication of results.

Disordered eating behaviours may be present in people with CF.^28^ Although concerns have been raised regarding the potential for intentional weight loss to exacerbate these behaviours, evidence from systematic reviews and RCTs in other populations with overweight and obesity suggests that structured behavioural weight management interventions do not worsen, and may improve, disordered eating symptoms.^29–31^ The development of this intervention was informed by behaviour change theory, including the Capability, Opportunity and Motivation – Behaviour model, Theoretical Domains Framework and OxFAB taxonomy.^32–34^ Behaviour change techniques targeted psychological capability (knowledge, skills and behavioural regulation), social and physical opportunity (environmental restructuring, social support) and reflective motivation (beliefs, goals and reinforcement). Habit theory was incorporated to support disruption of established cue-behaviour associations and formation of new habits.^35^ Key components include dietitian-led support to maintain motivation and address barriers, guidance on managing lapses and social situations, realistic goal setting with regular self-weighing and provision of a summary booklet and self-selected meal options tailored to participants’ socioeconomic circumstances. The intervention development process will be reported separately.

### Comparator

Participants allocated to the routine care will see a specialist CF dietitian as part of their multidisciplinary care, typically every 3 months, which is the standard of care in the UK.^36^ The dietitian may provide advice and support regarding weight management and behavioural changes if they feel it is required. Current clinical guidelines for people with CF recommend no intervention beyond healthful eating advice for those with overweight or obesity.^19–21^ To incentivise trial participation, the routine care group participants will be offered a one-off 30 min weight-loss consultation with a specialist CF dietitian at the end of the study.

### Schedule of study events

Table 1 provides a schedule of study events.

**Table 1:** Schedule of study events

Procedures	Assessments				6 months after last study visit	Up to 2 years after last study visit
	0 weeks	4 weeks	12 weeks	24 weeks		
	Screening/ baseline	Assessment 1	Assessment 2	Assessment 3		
Informed consent	x					
Eligibility assessment	x					
Demographic questionnaire	x					
Medical history	x					
Concomitant medications	x	x	x	x		
Randomisation	x					
EQ-5D-5L questionnaire	x			x		
AWESCORE questionnaire	x			x		
Weight and fat/fat-free mass	x	x	x	x		
Height	x					
Blood pressure	x			x		
Five times sit-to-stand	x	x	x	x		
Lung function	x	x	x	x		
Lipid profile	x			x		
HbA1c	x			x		
Adverse events assessments	x	x	x	x		
Qualitative interview with participants			x	x		
Qualitative interviews with staff				x		
Feedback questionnaire end of trial (all participants)				x		
Feedback questionnaire end of trial (intervention participants)				x		
Fidelity of intervention delivery		x	x	x		
Additional interview with intervention participants 6 months after the last visit (optional)					x	
Follow-up through medical notes						x

EQ-5D-5L, EuroQol 5-dimension 5-level.

### Baseline data collection

Participants will be asked to self-report their age, sex and ethnicity, relevant medical history, and all current medications, including CFTR modulators and corticosteroids.

### Physical measurements

All assessments will be conducted during a video call to allow researcher guidance. Height will be obtained from medical records or self-reported if unavailable. Participants will measure body weight barefoot and in light clothing using a calibrated digital scale and body composition (fat-free mass and fat mass) using a bioelectrical impedance analyser (BC-730 Tanita, Japan). Participants will be provided with standardised instructions to undertake measurements after voiding and under consistent conditions where possible, including in similar clothing. Body composition measurements will be considered exploratory and interpreted with appropriate caution given the known limitations of bioelectrical impedance analysis. Physical function will be assessed using the 5-times sit-to-stand test.^37^ Participants will measure their blood pressure at home using a validated monitor (Omron 10, Japan) following standard blood pressure measurement guidance.^38^ Spirometry will be performed according to British Thoracic Society guidelines^39^ using a home spirometer (MIR Spirobank Smart, Italy). Blood sample HbA1c and lipid profile will be assessed at baseline and 24 weeks using a home finger-prick blood sampling kit that participants will post to an established laboratory provider (Medichecks, UK). Where available, results obtained at the CF centre within 1 month of the baseline or 24-week visit will be used.

### Questionnaires

At baseline and end of study, the participants will be asked to complete the following health-related quality of life questionnaires. The European Quality of Life Five Dimension questionnaire and subscale (EQ-5D-5L)^40^ and the AWESCORE, a validated 10-item CF-specific quality of life instrument.^41^ At study completion, participants will complete a questionnaire to evaluate the acceptability of both the intervention and the study procedures.

### Long-term follow-up data

To provide early data on the sustainability of weight change and inform selection of longer-term outcomes for a definitive trial, we will collect routine clinical weight and lung function data for up to 2 years after completion of the 24-week study period. Participants will be asked for optional consent at enrolment for access to these routine data and to confirm that their CF centre may be contacted for data extraction where required.

### Outcomes

#### Primary feasibility outcomes

The primary objective of this study is to assess the feasibility of progression to a definitive RCT. The following progression criteria will determine whether to proceed to a full RCT without changes (green), proceed with amendments (amber) or not proceed (red) (table 2).

**Table 2:** Progression criteria for feasibility outcomes

Criterion	Measure	Green (progress)	Amber (progress with modifications)	Red (stop – do not proceed to definitive trial)
Recruitment	1a Rate (*n* of patients per site per month)	≥0.6	0.35–0.59	≤0.35
Recruitment	1b Total *N* participants recruited	≥30	16–29 (consider adding sites)	15
Engagement	Proportion of participants who attended ten or more sessions during the 24 weeks (60% of sessions) and at least one of the last three sessions.	≥60%	36%–59% (consider modifying based on process evaluation)	≤35%
Adherence	Proportion of participants in the intervention group with ≥5% wt loss at 12 weeks	≥50%	36%–49%	≤35%
Retention	Proportion of randomised participants completing a 24-week follow-up visit	≥75%	51%–74%	≤50%
Safety	Safety profile	Adjudicated by the Study Steering Committee, based on AE and SAE in the intervention arm which are related and unexpected		

AE, adverse event; SAE, serious AE.

- **Recruitment rate per month and overall recruitment:** assessed using the number of sites open, the total number of participants recruited, and the number of participants recruited per site.
- **Engagement rate:** assessed using the proportion of participants who attended ten or more sessions during the 24 weeks (60% of sessions), inclusive of at least one of the last three sessions.
- **Adherence rate:** assessed using the proportion of intervention participants with ≥5% wt loss at 12 weeks. The ≥5% wt loss threshold at 12 weeks reflects a clinically meaningful benchmark commonly used in weight management studies and provides an early signal of intervention response. Additional data will include attendance at scheduled dietetic appointments, self-reported adherence to the meal replacement/provided meals components and ordering data.
- **Retention rate:** assessed using the proportion of randomised participants completing a 24-week follow-up visit measured using data documented in the study notes at one time point.
- **Safety profile:** assessed using adverse events and serious adverse events in the intervention arm that are related and unexpected.

#### Exploratory outcomes

Exploratory outcomes detailed below will be collected to inform suitable outcomes for a future definitive RCT.

1. Body weight and body composition (weight, fat-free mass and fat mass) using provided scales (Tanita) at home at baseline, 4, 12 and 24 weeks.
2. Health-related quality of life using the EuroQol health questionnaire (EQ-5D-5L)^40^ and AWESCORE^41^ (CF-specific quality of life score) at baseline and 24 weeks.
3. Lung function (forced expiratory volume predicted (FEV1%) and forced vital capacity predicted (FVC%)) measured using home spirometry at baseline, 4, 12 and 24 weeks.
4. Physical function measured using the Five Times Sit to Stand Test^37^ observed on a video call at baseline, 4, 12 and 24 weeks.
5. Respiratory exacerbation rate recorded as adverse events during the study.
6. Blood pressure using home blood pressure monitors at baseline and 24 weeks.
7. Glycaemic control – HbA1c using a home blood test kit at baseline and 24 weeks.
8. Blood lipid profile (total cholesterol, HDL, LDL and non-fasting triglycerides) assessed at baseline and 24 weeks using a home blood test kit.
9. Adverse events in the intervention arm which are judged to be related and unexpected.

#### Process measures

The following process measures will be collected to inform any required changes to the intervention or trial design:

- Participant experience, assessed via end-of-trial questionnaires.
- Control group contamination, through qualitative interviews and end-of-study questionnaires, including assessment of whether routine care participants adopted weight loss strategies consistent with the intervention.
- Fidelity of delivery, assessed by JS reviewing recorded consultations against the intervention manual checklist.

### Retention strategies

The study has been designed to minimise burden to participants, with all visits conducted remotely and no requirement to attend additional appointments at the CF centre. This includes collection of home measurements, including blood samples. Participants will receive a £10 voucher for each completed study assessment, up to a maximum of £40, to compensate for the time spent completing study assessments.

### Adverse events

Participant safety will be monitored throughout the study. Adverse events and serious adverse events will be recorded at each study assessment and reviewed by the study team. As the intervention is considered low risk, adverse events are expected to be limited to those related to the underlying disease; however, any safety concerns arising during the study will be reviewed by the Study Steering Committee. Adverse events will be reported according to standard terminology.^42^ The safety reporting window will start from randomisation and continue until the 24 weeks of active study participation.

### Data management

Data will be managed in accordance with Good Clinical Practice, the Data Protection Act 2018 and the General Data Protection Regulation. Data will be entered directly onto electronic case report forms within a secure, web-based database (REDCap^43^ hosted by the Medical Sciences Division, University of Oxford). The database includes integrated query management functions, which will be monitored throughout and reviewed with the recruiting sites.

### Statistical analysis

The primary objective is to assess the feasibility of progression to a definitive RCT. Progression criteria will be summarised descriptively as counts and proportions. Exploratory outcomes will be summarised descriptively by study arm using an intention-to-treat approach including all randomised participants. No interim analyses are planned. As this is a feasibility study, we will explore between-group differences in secondary and exploratory outcomes. Estimates of effect will be interpreted cautiously as hypothesis generating and used to inform outcome selection and sample size calculation for a future definitive trial. Missing data will be summarised descriptively, and no imputation is planned. Qualitative findings will support interpretation of feasibility outcome data. Analyses will be conducted using R.^44^

## Patient and public involvement

An initial focus group with 7 individuals of varying ages with lived experience of CF and excess weight was conducted with support from the UK CF Trust patient involvement team. Participants expressed a preference for tailored support from a specialist CF dietitian, rather than referral to a generic weight management programme, and favoured a prescriptive dietary approach. Their input informed prioritisation and refinement of the research question.

A core group of 4 PPI participants was subsequently established and contributed to intervention development, including type of dietary intervention, study design and potential recruitment and retention strategies. They reviewed participant-facing materials, leading to revisions to improve clarity. One PPI member has joined the study steering committee. The PPI group will continue to contribute throughout the study, including advising on recruitment strategies, interpretation of findings and dissemination of results. They will also contribute to refining the intervention should it progress to a full RCT.

## Qualitative sub-study

All intervention participants will be offered the option to participate in semi-structured qualitative interviews at 12 or 24 weeks and all routine care participants at 24 weeks. These interviews will explore their acceptability of the intervention, overall trial experience, explore barriers and facilitators to adherence, and identify suggestions that could refine the intervention and trial should it progress to a full RCT. Interviews will also explore participants’ experiences of dietary change, including any psychological or social impacts related to eating behaviours, weight and body image. Participants will have the option to consent to be invited to participate in an additional interview 6 months after the end of their active study participation to provide further data on their experience following completion of the study.

Semi-structured interviews with 10 clinical staff involved in delivering the intervention will explore perceived barriers and facilitators to recruitment, delivery and potential integration of the intervention into routine clinical practice.

All interviews will be conducted by JS, a CF dietitian and developer of the intervention, who will not be routinely involved in intervention delivery or participants’ routine clinical care. Participants will be reassured that their responses will not affect their clinical care. Interviews will last approximately 30 minutes and be audio and video-recorded, transcribed verbatim and analysed using framework analysis, with researcher reflexivity considered throughout the analysis.^45^

## Study management and monitoring

Given the feasibility nature and scale of the study, no formal Data Monitoring Committee or stopping rules are planned. A study management group will be comprised of all named investigators, the study manager (JS) and relevant Oxford Respiratory Trials Unit personnel. It will oversee the day-to-day trial management and meet monthly to review progress and agree actions. An independent study steering committee will provide oversight, inclusive of data management and ethics aspects. It will consist of an academic respiratory consultant (chair), a CF dietitian and a patient representative.

## Ethics and dissemination

Substantial amendments to the protocol will be notified and reviewed by NHS Research Ethics Committee, the HRA and the Sponsor (University of Oxford) and the trial registry will be updated accordingly. Findings from this feasibility study will be submitted to a peer-reviewed journal and presented at conferences. Results will also be shared with participants and the wider public through the CF Trust. No specific post-trial care is planned, and participants will continue to receive routine clinical care throughout and following the study. De-identified participant data generated during the study may be made available on reasonable request to the corresponding author, subject to institutional approvals and data sharing agreements.

## Data Availability

No data are available.

## References

[1] Burgel P-R, Southern KW, Addy C (2024). Standards for the care of people with cystic fibrosis (CF); recognising and addressing CF health issues. J Cyst Fibros.

[2] Cystic Fibrosis Trust (2024). UK cystic fibrosis registry 2023 annual data report.

[3] Fajac I, Burgel PR, Martin C (2024). New drugs, new challenges in cystic fibrosis care. Eur Respir Rev.

[4] Middleton PG, Mall MA, Dřevínek P (2019). Elexacaftor-Tezacaftor-Ivacaftor for Cystic Fibrosis with a Single Phe508del Allele. N Engl J Med.

[5] Frost FJ, Peckham DG, Felton IC (2025). Managing an ageing cystic fibrosis population: challenges and priorities. Eur Respir Rev.

[6] Vavrina K, Griffin TB, Jones AM (2025). Evolving nutrition therapy in cystic fibrosis: Adapting to the CFTR modulator era. Nutr Clin Pract.

[7] Caley L, Peckham D (2022). Time to change course and tackle CF related obesity. J Cyst Fibros.

[8] Frost F, Nazareth D, Fauchier L (2023). Prevalence, risk factors and outcomes of cardiac disease in cystic fibrosis: a multinational retrospective cohort study. Eur Respir J.

[9] Archangelidi O, Cullinan P, Simmonds NJ (2022). Incidence and risk factors of cancer in individuals with cystic fibrosis in the UK; a case-control study. J Cyst Fibros.

[10] Adeniyi FB, Young T (2012). Weight loss interventions for chronic asthma. Cochrane Database Syst Rev.

[11] Peralta GP, Marcon A, Carsin A-E (2020). Body mass index and weight change are associated with adult lung function trajectories: the prospective ECRHS study. Thorax.

[12] Ryan DH, Yockey SR (2017). Weight Loss and Improvement in Comorbidity: Differences at 5%, 10%, 15%, and Over. Curr Obes Rep.

[13] Bruijn NRA, Wagenmakers MAEM, van Hoek M (2023). Bariatric surgery in a patient with cystic fibrosis and diabetes: A case report. J Cyst Fibros.

[14] Ahmed A, Ankireddypalli A, Harindhanavudhi T (2024). Glucagon-like peptide1 receptor agonist treatment of cystic fibrosis-related diabetes complicated by obesity: A cases series and literature review. J Clin Transl Endocrinol.

[15] Kumar S, Cobb R, Matson A (2026). Muscle Dysfunction and Bone Loss in a Woman With Cystic Fibrosis and Obesity Treated With Glucagon-Like Peptide 1 Agonist: A Case Report. Respirol Case Rep.

[16] Snowball JE, Flight WG, Heath L (2023). A paradigm shift in cystic fibrosis nutritional care: Clinicians’ views on the management of patients with overweight and obesity. J Cyst Fibros.

[17] Snowball J, Collins S, Smith C (2024). P330 Navigating the unknown: an international survey of current dietetic practices for overweight and obesity in people with cystic fibrosis. J Cyst Fibros.

[18] Anderson HL, Lynch V, Moore JE (2023). What is the Perceived Role of the Dietitian Amongst People with Cystic Fibrosis? Results of an International survey. Can J Diet Pract Res.

[19] van der Haak N, King SJ, Crowder T (2020). Highlights from the nutrition guidelines for cystic fibrosis in Australia and New Zealand. J Cyst Fibros.

[20] Wilschanski M, Munck A, Carrion E (2024). ESPEN-ESPGHAN-ECFS guideline on nutrition care for cystic fibrosis. Clin Nutr.

[21] Leonard A, Bailey J, Bruce A (2023). Nutritional considerations for a new era: A CF foundation position paper. J Cyst Fibros.

[22] Rowbotham NJ, Smith S, Elliott ZC (2023). A refresh of the top 10 research priorities in cystic fibrosis. Thorax.

[23] Snowball J, Adler A, Lally P (2026). EASE-CF: a randomised controlled feasibility trial of intentional weight loss in adults with cystic fibrosis. J Cyst Fibros.

[24] National Institute for Clinical E (2014). Obesity: identification, assessment and management.

[25] Lewis M, Bromley K, Sutton CJ (2021). Determining sample size for progression criteria for pragmatic pilot RCTs: the hypothesis test strikes back!. Pilot Feasibility Stud.

[26] Eldridge SM, Lancaster GA, Campbell MJ (2016). Defining Feasibility and Pilot Studies in Preparation for Randomised Controlled Trials: Development of a Conceptual Framework. PLoS One.

[27] Sealed Envelope Ltd. 2024 https://www.sealedenvelope.com/simple-randomiser/v1/.

[28] Powers KE, Bustos A, McCoy J (2025). Review of Disordered Eating Behaviors in Cystic Fibrosis. Life (Basel).

[29] Tsompanaki E, Koutoukidis DA, Wren G (2025). The impact of weight loss interventions on disordered eating symptoms in people with overweight and obesity: a systematic review & meta-analysis. EClinicalMedicine.

[30] Jebeile H, Libesman S, Melville H (2023). Eating disorder risk during behavioral weight management in adults with overweight or obesity: A systematic review with meta-analysis. Obes Rev.

[31] Tsompanaki E, Aveyard P, Park RJ (2025). An intensive weight loss programme with behavioural support for people with type 2 diabetes at risk of eating disorders in England (ARIADNE): a randomised, controlled, non-inferiority trial. Lancet Psychiatry.

[32] Michie S, Ashford S, Sniehotta FF (2011). A refined taxonomy of behaviour change techniques to help people change their physical activity and healthy eating behaviours: the CALO-RE taxonomy. Psychol Health.

[33] Michie S, Johnston M, Abraham C (2005). Making psychological theory useful for implementing evidence based practice: a consensus approach. Qual Saf Health Care.

[34] Hartmann-Boyce J, Aveyard P, Koshiaris C (2016). Development of tools to study personal weight control strategies: OxFAB taxonomy. *Obesity (Silver Spring*).

[35] Lally P, Chipperfield A, Wardle J (2008). Healthy habits: efficacy of simple advice on weight control based on a habit-formation model. Int J Obes (Lond).

[36] Cystic Fibrosis Trust (2024). Standards for the clinical care of children and adults with cystic fibrosis in the UK.

[37] Steffens D, Pocovi NC, Bartyn J (2023). Feasibility, Reliability, and Safety of Remote Five Times Sit to Stand Test in Patients with Gastrointestinal Cancer. Cancers (Basel).

[38] Excellence NIfHaC (2026). Hypertension in adults: diagnosis and management (NG136).

[39] British Thoracic Society (2013). A guide to performing quality assured diagnostic spirometry.

[40] Herdman M, Gudex C, Lloyd A (2011). Development and preliminary testing of the new five-level version of EQ-5D (EQ-5D-5L). Qual Life Res.

[41] Button BM, Wilson LM, Burge AT (2021). The AWESCORE, a patient-reported outcome measure: development, feasibility, reliability, validity and responsiveness for adults with cystic fibrosis. ERJ Open Res.

[42] Institute USDoHaHSNIoHNC (2017). Common terminology criteria for adverse events (CTCAE) version 5.0.

[43] Harris PA, Taylor R, Thielke R (2009). Research electronic data capture (REDCap)--a metadata-driven methodology and workflow process for providing translational research informatics support. J Biomed Inform.

[44] R Foundation for Statistical Computing (2024). R: a language and environment for statistical computing [program].

[45] Gale NK, Heath G, Cameron E (2013). Using the framework method for the analysis of qualitative data in multi-disciplinary health research. BMC Med Res Methodol.

